# Emotional and behavioral problems of 7-11 year old children in war-torn Nagorno–Karabakh region in Azerbaijan

**DOI:** 10.1192/j.eurpsy.2022.1121

**Published:** 2022-09-01

**Authors:** N. Osmanli, A. Babayev, I. Rustamov, K. Munir

**Affiliations:** 1 1 The Administration of the Regional Medical Divisions, Medical Services Department, Baku, Azerbaijan; 2 Khazar University, School Of Humanities And Social Sciences, Baku, Azerbaijan; 3 Azerbaijan Medical University, Department Of Psychiatry, Baku, Azerbaijan; 4 Harvard Medical School, Boston Children’s Hospital,developmental Medicine Center, Boston, United States of America

**Keywords:** Child mental health, Emotional and behavioral problems, Azerbaijan, armed conflict

## Abstract

**Introduction:**

The emotional, behavioral and psychosocial effects of chaotic environments following wars and armed conflicts in terms of exposure to trauma and displacement is well recognized. School-age children who are directly exposed to or witnessed negative effects of armed conflicts show an array of emotional and behavioral problems.

**Objectives:**

Our study aimed to examine the mental health conditions of children living in war and conflict zones and attending primary schools in Agdam.

**Methods:**

The study sample comprised of 617 children (mean age 8.9, SD 1.24; 50.7% female), residing in the conflict areas in the southwestern of Azerbaijan. The children were evaluated with the previously validated Azerbaijani version of the Strengths and Difficulties Questionnaire (SDQ) Teacher Form.

**Results:**

About a third of children (32.7%) had abnormal total scores, and a fifth (21.4%) were in borderline range. The SDQ subscale scores included emotional problems (19.4%); conduct problems (20.3%), hyperactivity/inattention (12.2%), peer relationship problems (31.1%), and pro-social behavior difficulties (13.1%). As a result, externalizing problem scores were higher in males (p<.001) and internalizing problems in females (p<.05). Due to correlation analysis, age is negatively and significantly related with externalising (p<.05), internalising (p<.01), and total difficulty (p<.05) scores.

**Conclusions:**

The findings of the study show that more than half of the children living in the war zone in Azerbaijan suffer from mental health problems and highlight the need for child mental health services and family supports in the region.

**Disclosure:**

No significant relationships.

